# Application of asymmetric flow field-flow fractionation (AF4) and multiangle light scattering (MALS) for the evaluation of changes in the product molar mass during PVP-b-PAMPS synthesis

**DOI:** 10.1007/s00216-018-1039-1

**Published:** 2018-04-16

**Authors:** Catalina Fuentes, Joel Castillo, Jose Vila, Lars Nilsson

**Affiliations:** 1 Food Colloids Group, Department of Food Technology, Engineering, and Nutrition, Faculty of Engineering, Lund University, PO Box 124, 22100 Lund, Sweden; 2 School of Chemistry, Faculty of Pure and Natural Science, Universidad Mayor de San Andres (UMSA), PO Box 330, Cota Cota 27 St., La Paz, Bolivia; 3 Department of Surgery, Biomedical Center, Group of Pancreatology, Faculty of Medicine, Lund University, 22184 Lund, Sweden; 4 Natural Product Laboratory, Hemisynthesis and Green Chemistry, Chemical Science Department, School of Pure and Natural Sciences FCPN, Universidad Mayor de San Andres (UMSA), PO Box 303, Ciudad Universitaria Cota Cota 27 St., La Paz, Bolivia

**Keywords:** Changes in molar mass, Synthesis, PVP-b-PAMPS, Asymmetric flow field-flow fractionation (AF4)

## Abstract

The use of polymers for the delivery of drugs has increased dramatically in the last decade. To ensure the desired properties and functionality of such substances, adequate characterization in terms of the molar mass (M) and size is essential. The aim of this study was to evaluate the changes in the M and size of PVP-b-PAMPS when the amounts of the synthesis reactants in the two-step radical reaction were varied. The determination of the M and size distributions was performed by an asymmetric flow field-flow fractionation (AF4) system connected to multiangle light scattering (MALS) and differential refractive index (dRI) detectors. The results show that the M of the polymers varies depending on the relative amounts of the reactants and that AF4-MALS-dRI is a powerful characterization technique for analyzing polymers. Using AF4, it was possible to separate the product of the first radical reaction (PVP-CTA) into two populations. The first population had an elongated, rod-like or random coil conformation, and the second had a conformation corresponding to homogeneous spheres or a microgel structure. PVP-b-PAMPS had only one population, which had a rod-like conformation. The molar masses of PVP-CTA and PVP-b-PAMPS found in this study were higher than those reported in previous studies.

## Introduction

In recent decades, the use of polymers as a means of drug delivery has increased. Polymeric micelles have become an attractive option because of their capability to protect and extend the lifetime of a drug. Polymeric micelles can be divided in two categories: hydrophobically assembled micelles and polyion complex micelles, where cationic and anionic segments form polyion complex micelles. In the present study, PVP (poly-vinylpyrrolidone) acts as the hydrophilic nonionic segment and PAMPS (poly-2-acrylamido-2-methyl-1-propanesulfonic acid) as the polyanion segment for the synthesis of PVP-b-PAMPS [1]. In this sense, the use of PVP in polymeric micelles has been demonstrated to form microgels, which showed no toxicity when they were used in saline solutions as a blood extender [2, 3]. In addition, PVP-b-PAMPS in combination with PEG-b-P4VP could be used as a drug delivery system [4].

The physical and mechanical properties of polymers depend on the molecular architecture, molar mass (M), size, and distributions [5]. The two fundamental properties, the size and M of polymers, can be experimentally determined in a number of ways. Batch-mode determinations can be performed with techniques such as viscometry, light scattering, and osmometry. These techniques provide differently weighted size or M averages, but little or no information is obtained on the size or M distributions. To generate an accurate characterization, separation methods connected to various detectors are typically used. The most commonly used separation method for polymers is size exclusion chromatography (SEC). In this technique, the size and M can be estimated either with a standard calibration curve or by absolute determination of both the M and average size by utilizing online multiangle light scattering (MALS) in combination with concentration determination via, for instance, differential refractive index (dRI) detection. SEC has some limitations, which include the adsorption of sample components to the column, the degradation of large species due to shear forces in the column, and the co-elution effects that can arise, for instance, from the presence of branches in the polymers [6–8]. This can produce abnormal elution effects that make the determination of the M distribution difficult or impossible [8, 9].

Some of the drawbacks of SEC can be avoided by instead utilizing asymmetric flow field-flow fraction (AF4) [10–12]. AF4 has been shown to be suitable for the analytical separation of macromolecules and aggregated structures, and interest in the method has increased in recent years [13, 14]. The separation method is based on the longitudinal laminar flow of a carrier liquid through a separation channel in combination with a crossflow in a perpendicular direction over the channel. The crossflow forces the sample components towards the ultrafiltration membrane, which acts as an accumulation wall. At the accumulation wall, the components are confined to a thin concentrated layer. In Brownian mode AF4, the crossflow-induced transport is counteracted by the diffusion of the sample components and at steady state, a concentration profile is established in the sample zone. The result is that sample components with a higher diffusion coefficient (D), on average, will be distributed farther away from the accumulation wall than components with a lower D. As the flow profile along the separation channel is parabolic, the components distributed farther away from the accumulation wall will travel faster downstream and, thus, size separation is achieved. One important parameter of the ultrafiltration membrane, which makes up the accumulation wall, is the cut-off, which must be sufficiently low to keep the sample in the channel as sample components smaller than the cut-off may permeate through the membrane. An AF4 system is most suitably connected to a MALS detector and a concentration detector (such as dRI) in a similar way as an SEC system for the determination of size and M distributions.

As mentioned above, the adequate characterization of polymers is essential to ensuring the desired properties and functionality of the polymers. In this study, the aim was to apply AF4-MALS-dRI in order to evaluate the changes in M and size when three different amounts of the reactants were varied in the two consecutive radical reactions that occur during the synthesis of PVP-b-PAMPS.

## Materials and methods

### Materials

1-Vinyl-2-pyrrolidinone (VP), 2,2'-azobis(2-methylpropionitrile) (AIBN), 2-dodecylsulfanylthiocarbonylsulfanyl-2-methyl propionic acid (DMP), tetrahydrofuran (THF), 2-acrylamido-2-methyl-1-propanesulfonic acid (AMPS), *N,N*-dimethylformamide (DMF), acetone, and diethyl ether were purchased from Sigma-Aldrich (Sigma Aldrich, MO, USA).

### Methods

#### Synthesis of PVP-b-PAMPS

**Fig. 1:**
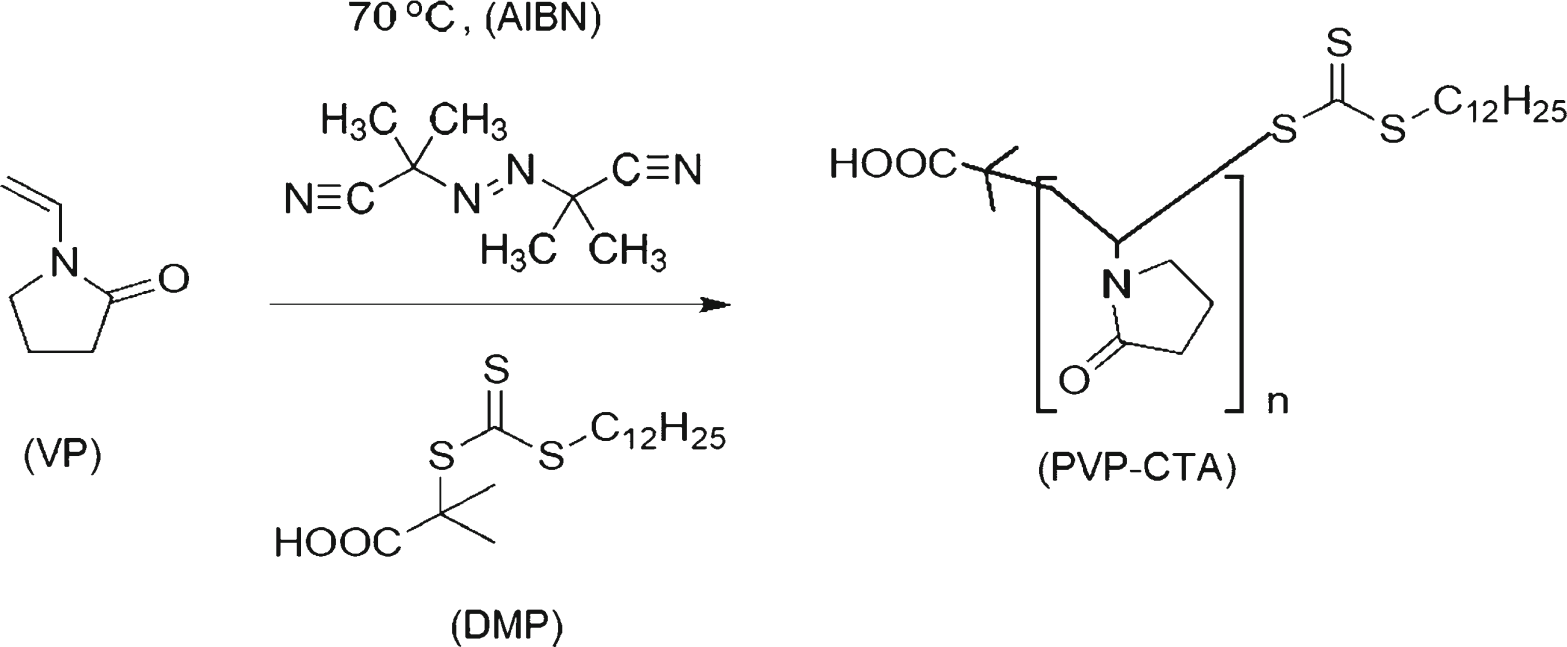
First radical reaction: 1-vinyl-2-pyrrolidone (VP), 2,2'-azobis(2-methylpropionitrile) (AIBN), and 2-dodecylsulfanylthiocarbonylsulfanyl-2-methyl propionic acid (DMP)

**Fig. 2:**
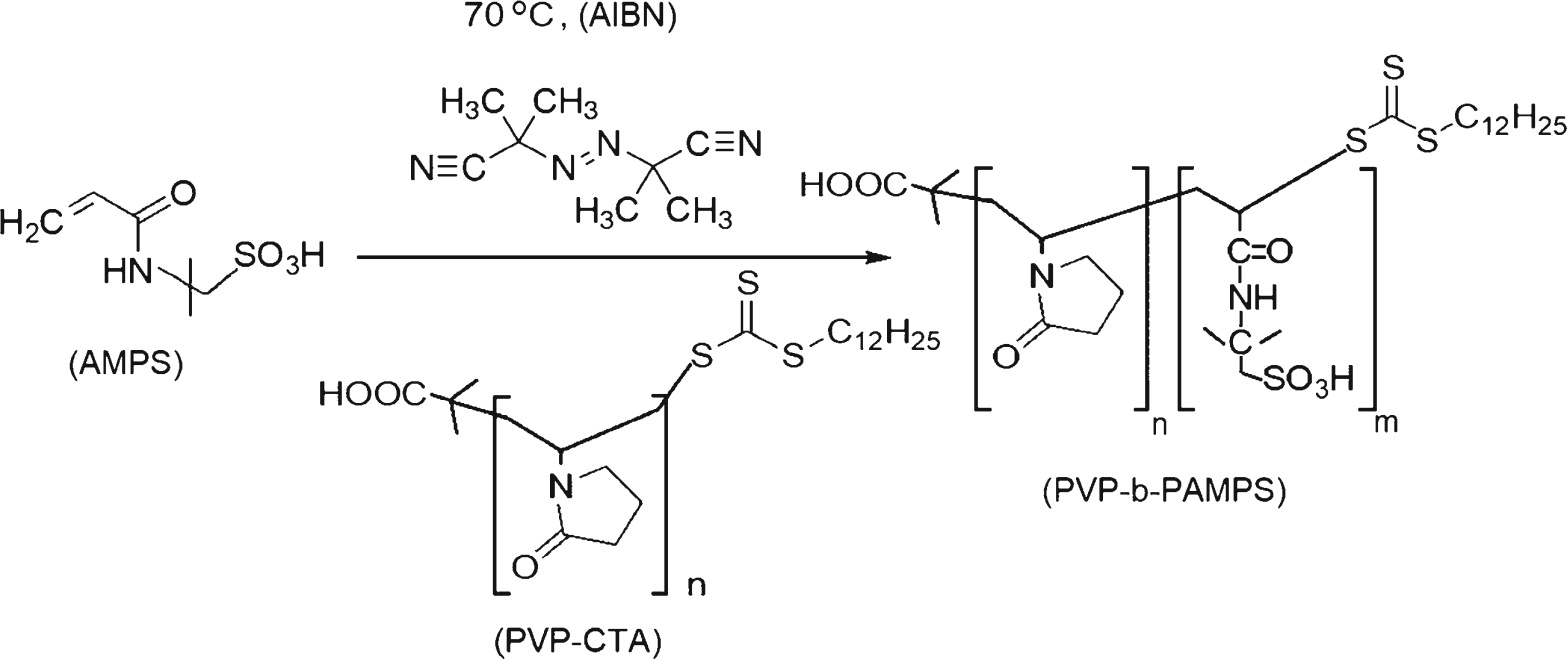
Second radical reaction: PVP-CTA, 2,2'-azobis(2-methylpropionitrile) (AIBN), 2-acrylamido-2-methyl-1-propanesulfonic acid (AMPS)

Poly (*N*-vinyl-pyrrolidone)-block-poly(2-acrylamido-2-methyl-1-propanesulfonic acid) (PVP-b-PAMPS) was achieved through two consecutive radical reactions with 1-vinyl-2-pyrrolidone (VP) and 2-acrylamido-2-methyl-1-propanesulfonic acid (AMPS), respectively. Reversible addition-fragmentation chain transfer (RAFT) using DMP and PVP, respectively, as the chain transfer agent (CTA) controlled the radical reactions. The synthesis is shown schematically in Figs. 1 and 2.

The synthesis of PVP-b-PAMPS was performed as described elsewhere [1, 4], with some modifications. To evaluate the changes in M, the amounts of the reactants were varied in each radical reaction. An overview of the samples and a description of the variation of reactants amounts is shown in Table 1.

**Table 1:** Overview of the polymer samples and the variation in the amounts of reactants in the radical reactions

Samples of the first radical reaction	VP		AIBN		DMP		THF	Relative level of variation^a^
	(g)	(mmol)	(g)	(mmol)	(g)	(mmol)	mL	mol %
MPC-4	8.40	75.60	0.007	0.043	***0.047***	***0.13***	8	100
MPC-5	8.40	75.60	0.007	0.043	***0.091***	***0.25***	8	194
MPC-6	8.40	75.60	0.007	0.043	***0.182***	***0.50***	8	388
MPC-18	***0.05***	***0.46***	0.007	0.043	0.078	0.21	8	100
MPC-19	***0.10***	***0.92***	0.007	0.043	0.078	0.21	8	200
MPC-20	***0.20***	***1.84***	0.007	0.043	0.078	0.21	8	400
MPC-21	8.40	75.60	***0.036***	***0.217***	0.078	0.21	8	100
MPC-22	8.40	75.60	***0.071***	***0.429***	0.078	0.21	8	198
MPC-23	8.40	75.60	***0.140***	***0.851***	0.078	0.21	8	392
Samples of the second radical reaction	PVP-CTA		AIBN		AMPS		DMF	Relative level of variation^a^
	(g)	(mmol)	(g)	(mmol)	(g)	(mmol)	mL	mol %
MPC-24	1.607	0.016	0.007	0.043	***0.114***	***0.550***	15	100
MPC-25	1.607	0.016	0.007	0.043	***0.225***	***1.084***	15	197
MPC-26	1.607	0.016	0.007	0.043	***0.446***	***2.131***	15	387
MPC-27	***1.039***	***0.010***	0.007	0.043	4.530	21.860	15	100
MPC-28	***2.040***	***0.020***	0.007	0.043	4.530	21.860	15	196
MPC-29	***3.150***	***0.031***	0.007	0.043	4.530	21.860	15	303
MPC-30	1.607	0.016	***0.034***	***0.209***	4.530	21.860	15	100
MPC-31	1.607	0.016	***0.058***	***0.351***	4.530	21.860	15	167
MPC-32	1.607	0.016	***0.116***	***0.708***	4.530	21.860	15	338

^a^The mol % was calculated in relation to the lowest addition amount in the synthesis

The numbers in italics indicate the amount of each reactant in the radical reaction

In the first radical reaction, VP, AIBN, DMP (in the amount indicated in Table 1), and 8 mL of tetrahydrofuran (THF) were mixed together and purged with nitrogen for 30 min. The reaction tube was sealed and placed in a water bath at 70 °C for 12 h. The reaction mixture was cooled in an ice bath. Then, the reaction mixture was poured into diethyl ether under stirring. The largest particles were cut into smaller pieces to ensure better contact with the organic solvent. The solid was filtered out and mixed with diethyl ether under stirring for 10 min. After filtration, the solid was dried under vacuum to constant mass.

In the second radical reaction, PVC-CTA, AIBN, and AMPS, in the amounts indicated in Table 1, were dissolved in 15 mL of *N,N*-dimethylformamide (DMF). The solution was purged with nitrogen for 30 min, and the polymerization was started by heating at 70 °C for 12 h. After completion of the reaction, the polymeric product was precipitated in acetone:diethyl ether (70:30, v/v). The largest particles of the polymer were cut into small pieces to increase the surface interaction with the solvent. The mixture was filtered, and the solid was remixed with acetone:diethyl ether (70:30, v/v) under stirring for 10 min and filtered. The obtained solid was dried in a vacuum to constant mass. Finally, once the product was dried, the required aqueous solutions for AF4 were prepared as described below.

#### ^1^H NMR and FTIR

The NMR spectra were obtained using a Bruker ARX400 spectrometer (Bruker, Leiderdorp, Nederland) operated at 400 MHz. Dimethyl sulfoxide-d_6_ (DMSO-d_6_), purchased from Sigma-Aldrich (Sigma Aldrich, MO, USA), was used to prepare the samples. For PVP-CTA: ^1^H NMR (DMSO-d_6_) δ 9.5 – 10.0 (*br s*, COO**H**), 3.0 – 4.0 (*br m*, C**H**_2_-N and C**H**-N, pyrrolidinone unit), 2.5 – 1.2 (*br m*, C_12_**H**_25_, C**H**_3_ β-carboxylic). For, PVP-b-PAMPS: ^1^H NMR (DMSO-d_6_) δ 8.0 (*br s*, N**H**), 4.7 – 5.3 (*br m*, N-C**H**_2_, N-CO-C**H**, SO_3_H-C**H**_2_), 2.9 – 3.2 (*br s*, C**H**_2_-N, pyrrolidinone unit), 2.8 (*br m*, N-CO-CHC**H**_2_), 2.6 (*br m*, C**H**_2_-CO-N, pyrrolidinone unit), 2.4 (*br m*, CH_2_C**H**_2_CH_2_ (pyrrolidinone unit), 1.5 – 2.2 (*br m*, C**H**_3_ β-carboxylic and CH_3_ β-amido group (sulfonic unit) and C**H**_2_CH-N (pyrrolidinone unit) and 1.0 – 1.5 (*br m*, C_12_**H**_25_).

The FTIR spectra were recorded using an IFS 125HR FT–IR spectrometer (Bruker Optics Inc., 40 Manning Road, Billerica, MA, USA). Potassium bromide (KBr) used for the sample pellets was provided by Sigma-Aldrich (MO, USA). For PVP-CTA: IR (KBr) cm^-1^ 3300–2500 (*m*, O–H, carboxylic acid), 3000–2850 (*m*, C–H stretching alkanes), 1760–1690 (*s*, C=O stretching carboxylic acid and pyrrolidinone unit), 1335–1250 (*s*, C–N, stretching, amides), 1320–1000 (*s*, C–O stretching, carboxylic acid). For, PVP-b-PAMPS: IR (KBr) cm^-1^ 3300–2500 (*m*, O–H, carboxylic acid), 3000–2850 (*m*, C–H stretching, alkanes), 3400–3250 (*m*, N–H stretching, 2° amines), 1760–1690 (*s*, C=O stretching carboxylic acid and pyrrolidinone unit), 1345 (*s*, S=O stretching, sulfonic acid), 1335–1250 (*s*, C–N stretching, amines), 1320–1000 (*s*, C–O stretching, carboxylic acid), and 1200–1050 (*s*, C=S stretching, thiocarbonyl group).

#### Measurement of the specific refractive index increment (dn/dc)

The specific refractive index increment (dn/dc) was measured using an Optilab T-rEX differential refractive index detector (dRI) (Wyatt Technology, Dernbach, Germany) operating at a wavelength of 658 nm and 35 °C. The flow rate was adjusted to 0.5 mL/min using an Agilent 1100 series isocratic pump (Agilent Technologies, Waldbronn, Germany) with an in-line vacuum degasser and connected to a Rheodyne manual injector (Kinesis, St Neots, UK) with a 1 mL sample loop. Between the pump and the manual injector, a polyvinylidene fluoride membrane with a 100 nm pore size (Millipore Corp., Bedford, MA, USA) was placed to ensure that particle-free carrier entered the system. To verify the calibration of the cell in the dRI detector, an assay was performed using pre-prepared standard NaCl solutions (Wyatt Technology, Dernbach, Germany), injecting eight samples with different concentrations (0.1–5.0 mg/mL concentration range). For analysis of the polymer samples, a series of six different concentrations was injected for each sample. The sample solutions were prepared by dissolving the dried polymer in carrier liquid (1 mg/mL). The composition of the carrier liquid was 10 mM NaNO_3_ (AppliChem, A3125, Darmstadt, Germany) and 0.02% NaN_3_ (BDH, 10369, Poole, UK), dissolved in Milli–Q water. The remaining five solutions in the 0.1–0.8 mg/mL concentration range were prepared by dilution of the 1 mg/mL solution before injection. The samples were filtered through a 0.20 μm cellulose acetate filter (VWR syringe filter, 25 mm diameter, Leuven, Belgium). Each analysis was performed in triplicate, and the dn/dc value was determined from the slope of a plot of dRI against the concentration. The concentration used was corrected for the moisture content of the sample, which was determined gravimetrically using a moisture analyzer (MAC 110/WH, Radwag, Radom, Poland).

#### Asymmetric flow field-flow fractionation (AF4)

The polymer samples were analyzed with AF4-MALS-dRI using an Eclipse 3+ system (Wyatt Technology, Dernbach, Germany) connected to a Dawn Heleos II (MALS) detector (Wyatt Technology, Dernbach, Germany) and an Optilab T-rEX differential refractive index detector (dRI) (Wyatt Technology, Dernbach, Germany). Both detectors operated at a wavelength of 658 nm. An Agilent 1100 series isocratic pump (Agilent Technologies, Waldbronn, Germany) with an in-line vacuum degasser and an Agilent 1100 series autosampler delivered the carrier flow and handled the sample injection into the AF4 separation channel. Between the pump and the channel, a polyvinylidene fluoride membrane with a 100 nm pore size (Millipore Corp., Bedford, MA, USA) was placed to ensure that particle-free carrier entered the channel. The AF4 channel was a long separation channel (Wyatt Technology, Dernbach, Germany) with trapezoidal geometry (tip-to-tip length of 26.0 cm and inlet and outlet widths of 2.15 and 0.6 cm, respectively) and with a nominal thickness of 350 μm. The ultrafiltration membrane forming the accumulation wall was a regenerated cellulose membrane (RC) with a nominal cut-off of 10 KDa (Merck Millipore, Bedford, MA, USA). Validation of the performance of the AF4 system and the experimentally determined channel thickness was performed with bovine serum albumin (BSA, Sigma, A4378, St, Louis, MO, USA) solution (1 mg/mL, w/v) according to the procedure described in the literature (MATLAB-based software: FFFHydRad 2.2) [15, 16]. The actual channel height was determined to be 277 μm. The composition of the carrier liquid was 10 mM NaNO_3_ (AppliChem, A3125, Darmstadt, Germany) and 0.02% NaN_3_ (BDH, 10369, Poole, UK), dissolved in Milli-Q water.

The separation method used a constant detector flow of 1 mL/min. Injection into the channel was performed with a flow rate of 0.2 mL/min for 6 min. The sample volume injected into the channel was between 80 and 120 μL for an injected sample mass of approximately 80–120 μg. The injected amount was optimized in order to ensure that no overloading occurred, i.e., the retention time was independent of the amount injected. After injection, a 4 min focusing/relaxation step was performed prior to elution with a focus flow identical to the initial crossflow. The crossflow rate was programmed to decay exponentially using the following equation:

1 $$ {Q}_c(t)={Q}_c(0){e}^{\left(-\frac{\mathit{\ln}2}{t_{1/2}}t\right)} $$

where *Q*_*c*_ *(t)* is the crossflow rate as a function of time *t* after elution begins, *Q*_*c*_ *(0)* is the initial crossflow rate, and t_1/2_ is the half-life of the decay. For all samples, the elution began with an initial crossflow of 2.4 mL/min, which decreased exponentially over time to 0.12 mL/min, t_1/2_ = 6 min, and then remained constant for 10 min. Finally, the channel was flushed without any crossflow for 10 min before the next analysis. The recorded MALS and dRI data were processed using Astra software (ver. 6.1.5.22, Wyatt Technology). The M and r_rms_ were obtained from fitting the MALS data using the Berry method [17, 18] by performing a first order fit to the data obtained at scattering detectors 6–17 (angles 42.8°–152.5°). The specific refractive index (dn/dc) calculated for each sample as described above was used, and the second virial coefficient was assumed to be negligible. Assuming homogeneous distribution of mass and a spherical shape, the apparent density was calculated from the determined M and r_rms_ distributions [19]. The apparent density for component *I* of the sample *ρ*_*i*_ is given in the following equation:

2 $$ \widehat{\rho_i}=\frac{M_i}{V{\left({r}_{rms}\right)}_i}\cdotp q $$

where *M* is the molar mass, *V* is the volume, and *q* is a scaling constant relating the physical radius of a sphere and *r*_*rms*_. The mass recovery was determined from the ratio of the mass eluted from the separation channel (integration of the dRI signal) to the injected mass.

## Results and discussion

### ^1^H NMR and FTIR spectroscopy

The ^1^H NMR and IR data were used to confirm the chemical structures of the synthesized PVP-CTA and PVP-b-PAMPS polymers. For PVP-CTA, ^1^H NMR analysis showed a peak at 10.0–9.5 ppm confirming the presence of a carboxylic acid proton. The peaks observed at 4.0–3.0 ppm represent the protons on the carbon α to the nitrogen atom in the pyrrolidinone group. The rest of the protons, namely, the lipidic tail, the protons on the carbon γ to the carboxylic group, and the other protons contained in the pyrrolidinone group, are included in the peaks between 2.5 and 1.2 ppm. IR analysis showed the O-H carboxylic acid group at 3300–2500 cm^-1^. The peaks corresponding to the lipidic tail, and the β and γ carbons to the carbonyl group (sp^3^ carbons), were found at 3000–2850 cm^-1^. The peaks at 1760–1690 cm^-1^ represented the C=O stretching of the carboxylic acid and pyrrolidinone, and the C-N stretching of aromatic amines and the C-O stretching of the carboxylic acid were observed at 1335–1250 and 1320–1000 cm^-1^, respectively. Thiocarbonyl stretching of C=S was found at 1200–1050 cm^-1^.

For PVP-b-PAMPS, ^1^H NMR analysis showed a peak at 8.0 ppm associated with the proton on the nitrogen of the amide group. The signals for the protons on the carbons α to the nitrogen atom in the pyrrolidine-2-one group and the carbon α to the sulfonic group were found at 3.0–2.5 ppm. The peaks belonging to the protons on the carbon γ to the sulfonic group, the remaining proton on the pyrrolidine-2-one group, and the protons on the lipidic chain were observed at 2.5–1.0 ppm. The IR spectrum showed the amido group peak at 3400–3250 cm^-1^. A peak related to the O-H functionality on the carboxylic acid group was observed at 3300–2500 cm^-1^. The sp^3^ carbons contained in the lipidic tail and the β and γ carbons to the carbonyl group were associated with the 3000–2850 cm^-1^ peak. The signal located at 1760–1690 cm^-1^ was assigned to the C=O stretching of the carboxylic acid and pyrrolidinone unit. The sulfonic acid S=O stretching was found at 1345 cm^-1^. The C-N stretching of aromatic amines, C-O stretching of the carboxylic acid, and C=S stretching of the thiocarbonyl group were observed at 1335–1250 (s), 1320–1000 (s), and 1200–1050 (s), respectively.

### Determination of dn/dc

**Fig. 3:**
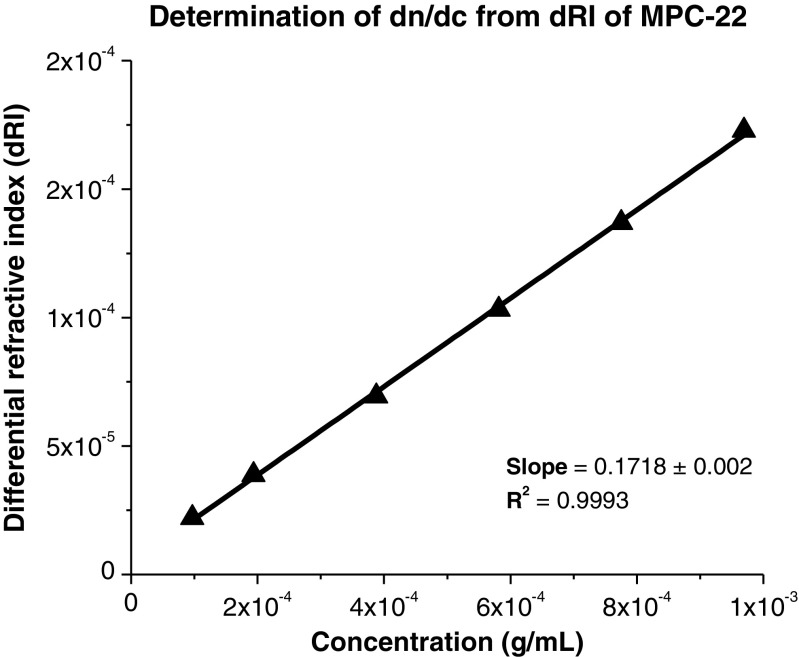
dn/dc Values for sample MPC-22. Average differential refractive index (dRI) versus concentration (g/mL)

Figure 3 shows a representative plot of the differential refractive index versus concentration. The results are an average of three replicate measurements. The values of the specific refractive index increment (dn/dc) for all the samples are shown in Table 2. Samples MPC-4 to MPC-23, which correspond to the product of the first radical reaction (Fig. 1), show similar dn/dc values that are not significantly different according to Tukey’s test.

**Table 2:** Summary of the results for the moisture content and dn/dc values, obtained from the differential refractive index (dRI) versus concentration analyses

Samples of the first radical reaction	Moisture content (%)^a^	dn/dc (mL/g)^b^	Error _dn/dc_ (mL/g)	Fit R^2^
NaCl	-------	0.174	0.001	1.000
MPC-4^c^	4.2 ± 0.0	0.177	0.002	0.999
MPC-5^c^	2.6 ± 0.1	0.176	0.002	0.998
MPC-6 ^c^	4.5 ± 0.2	0.171	0.002	0.999
MPC-18 ^c^	1.4 ± 0.3	0.177	0.001	0.999
MPC-19 ^c^	0.7 ± 0.1	0.175	0.002	0.999
MPC-20 ^c^	2.5 ± 0.1	0.174	0.001	0.999
MPC-21 ^c^	3.3 ± 0.1	0.171	0.002	0.999
MPC-22 ^c^	3.1 ± 0.1	0.172	0.001	0.999
MPC-23 ^c^	1.8 ± 0.0	0.171	0.001	0.999
Samples of the second radical reaction	Moisture content (%)^a^	dn/dc (mL/g)^b^	Error _dn/dc_ (mL/g)	Fit R^2^
MPC-24 ^d^	5.9 ± 0.4	0.138	0.001	1.000
MPC-25 ^d^	3.3 ± 0.2	0.138	0.001	0.999
MPC-26 ^e^	8.3 ± 0.0	0.147	0.001	0.999
MPC-27 ^d^	6.7 ± 0.2	0.138	0.001	1.000
MPC-28 ^d^	6.4 ± 0.2	0.138	0.001	1.000
MPC-29 ^e^	7.9 ± 0.2	0.132	0.001	1.000
MPC-30 ^d^	8.9 ± 0.2	0.142	0.001	0.999
MPC-31 ^e^	9.2 ± 0.2	0.144	0.001	1.000
MPC-32 ^d^	9.7 ± 0.4	0.140	0.001	1.000

^a^Standard deviation values (±) based on three replicates measurements

^b^All dn/dc values were corrected for the moisture content

^c^dn/dc for all samples during the first radical reaction; they were not significantly different from one another according to Tukey’s test (critical mean difference = 0.0073 mg/mL, α = 0.05) based on triplicate analysis

^d^dn/dc for all samples during the second radical reaction; they were not significantly different from one another according to Tukey’s test (critical mean difference = 0.0037 mg/mL, α = 0.05) based on triplicate analysis

^e^dn/dc for all samples during the second radical reaction; the sample was significantly different from the rest of samples according to Tukey’s test (critical mean difference = 0.0037 mg/mL, α = 0.05) based on triplicate analysis

For samples MPC-24 to MPC-32, corresponding to the product of the second radical reaction (Fig. 2), only MPC-29 was significantly different from all other samples (Tukey’s test). MPC-26 and MPC-31 were not significantly different from each other but were different from the rests of the samples. For the determination of the molar mass, the individual values of dn/dc of each sample were used.

### Molar mass, size, and conformation

AF4-MALS-dRI was used to determine the weight-average molar mass (M_w_) and z-average root-mean-square radius (r_rms_). The analysis was performed in triplicate. A summary of the results is shown in Table 3. Values of the separation channel recoveries for the first radical reaction were ≥76%, and those for the second radical reaction were ≥101%, which means that all or a large amount of the injected sample was analyzed without a major loss of components.

**Table 3:** Average values obtained from the AF4-MALS-dRI analyses

Sample of the first radical reaction	M_w_ range (10^5^g/mol)^a^	M_w_ (10^5^g/mol)^b^	r_rms_ (nm)^b^	r_rms_/r_hyd_^c^	Mass recovery – 10 kDa (%)^d^
MPC-4	0.1–2.0	0.40	14.9	2.0	82
MPC-5	0.1–2.1	0.39	15.2	2.0	76
MPC-6	0.1–1.7	0.36	15.0	2.3	80
MPC-18	0.1–7.4	1.21	20.9	1.1	103
MPC-19	0.1–17.0	1.76	23.5	1.0	102
MPC-20	0.1–2.0	0.42	12.4	1.7	81
MPC-21	0.1–6.4	0.57	13.4	1.4	91
MPC-22	0.1–1.6	0.39	11.3	1.7	85
MPC-23	0.1–1.4	0.32	12.1	1.3	76
Sample of the second radical reaction	Mw range (10^5^g/mol)^a^	M_w_ (10^5^g/mol)^b^	r_rms_ (nm)^b^	r_rms_/r_hyd_^c^	Mass recovery – 10 kDa (%)^d^
MPC-24	0.2–17.0	3.37	52.4	2.6	105
MPC-25	0.1–12.0	2.30	45.1	2.8	101
MPC-26	0.1–6.3	1.82	40.9	2.9	102
MPC-27	0.2–13.0	3.02	49.6	2.7	107
MPC-28	0.1–14.0	3.35	53.5	2.6	106
MPC-29	0.1–14.0	3.54	54.6	2.4	105
MPC-30	0.1–14.0	3.16	52.9	2.5	101
MPC-31	0.1–14.0	2.64	50.2	2.6	107
MPC-32	0.1–10.0	2.59	48.4	2.6	107

^a^M_w_ range is the molar mass range upon which the M_w_ is based

^b^M_w_ is the weight-average molar mass, and r_rms_ is the z-average root-mean-square radius

^c^The ratio is the quotient between r_rms_ and r_hyd_ as the conformational parameter

^d^The mass recovery was determined from the ratio of the mass eluted from the separation channel (integration of the dRI signal) to the injected mass on dry basis

Table 3 shows that the size of the polymers from the first radical reaction were relatively small, i.e., r_rms_ from 11 to 23 nm, and samples from the second radical reaction were somewhat larger, i.e., r_rms_ from 41 to 55 nm. It can be observed that the M_w_ values of PVP-CTA (3.2·10^4^ to 1.8·10^5^ g/mol) and PVP-b-PAMPS (1.8·10^5^ to 3.5·10^5^ g/mol) from the first and second radical reactions, respectively, obtained by AF4, were larger than those obtained by SEC in previous studies (3.3·10^4^ g/mol for PVP-CTA and 1.16·10^5^ g/mol for PVP-b-PAMPS) [1, 4]. The trend of obtaining higher values for M from AF4 than from SEC is commonly observed for large polymers. This trend is often due to the shear degradation in SEC. However, no information regarding the SEC columns was given in the previous studies [1, 4]. Thus, conclusions regarding the differences in the separation techniques are not possible to make.

**Fig. 4:**
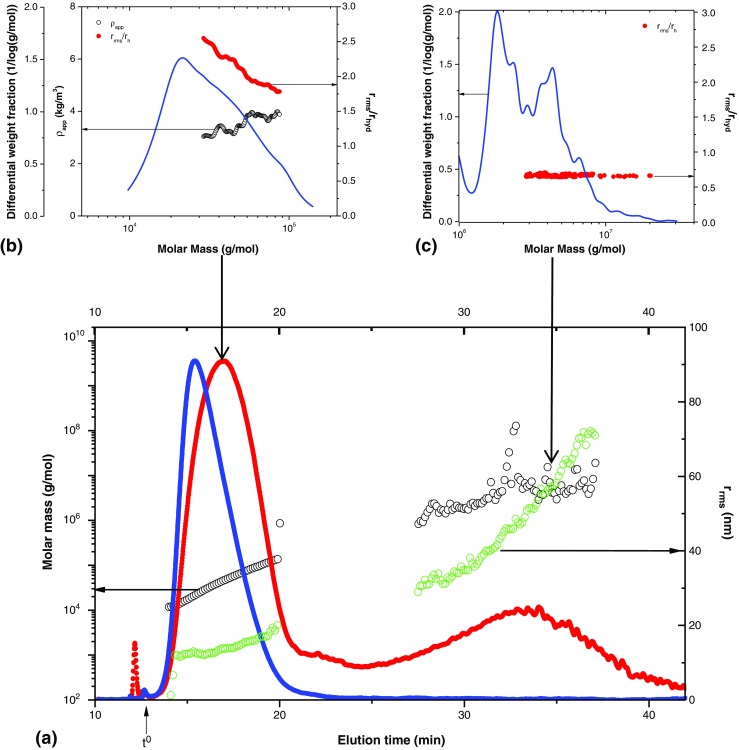
AF4 fractogram for sample MPC-5. (**a**) Molar mass distribution M (○), root- mean-square radius distribution r_rms_ (), Rayleigh ratio from MALS at 90^0^ () and differential refractive index dRI () versus the elution time (min). (**b**) Differential weight fraction 1/log(g/mol) (), apparent density ρ_app_ (○) and the ratio between the root- mean-square radius r_rms_ and hydrodynamic radius r_hyd_ as a conformational parameter versus the molar mass (g/mol) for the first population. (**c**) Differential weight fraction 1/log(g/mol) and the ratio between the root- mean-square radius r_rms_ and hydrodynamic radius r_hyd_ as a conformational parameter versus the molar mass (g/mol) for the second population

Figure 4 shows a representative AF4 fractogram for samples from the first radical reaction (MPC-4 to MPC-23) to yield PVP-CTA. The result shows the presence of two main populations (Fig. 4a). In previous studies, where the polymer was synthesized under similar conditions, the second, larger-size population was not reported in the SEC results [1, 4]. As mentioned above, no information about the SEC column was given in these studies. Hence, it is not possible to know whether the result could be an artifact of the analysis. The results in Fig. 4 also show that the populations have different conformations. The first population has different conformational properties, which scale strongly with increasing M (as shown by a decrease in r_rms_/r_hyd_ with an increase in M). The lower-M species (M of approximately 2.5–5∙10^4^ g/mol, Fig. 4b) have r_rms_/r_hyd_ = 2.0 to 2.6, corresponding to an elongated or rod-shaped conformation [20]. In the same population, the somewhat higher-M species (M of approximately 5–10∙10^4^ g/mol, Fig. 4b) have r_rms_/r_hyd_ = 1.7 to 2.0, which suggests a more flexible conformation, i.e., a random coil [20]. For the second and later-eluting population (see Fig. 4c), r_rms_/r_hyd_ shows no scaling with M and has a value of approximately 0.7. It is not possible to draw conclusions regarding the conformation from this value, as the error in the determination of r_rms_/r_hyd_ is approximately ± 0.1. Hence, r_rms_/r_hyd_ = 0.7 ± 0.1 could correspond to both a microgel structure (r_rms_/r_hyd_ < 0.7) [21] or to a homogeneous-mass spherical object (r_rms_/r_hyd_ = 0.775). As the species are substantially larger and have very different behaviors that scale constantly with M, these results suggest a uniformity in conformation with increasing M, which in turn suggests that the species are supra-molecular aggregates. It should be noted that the second population represented a very small fraction of the entire sample (i.e., a low dRI signal, Fig. 4a).

**Fig. 5:**
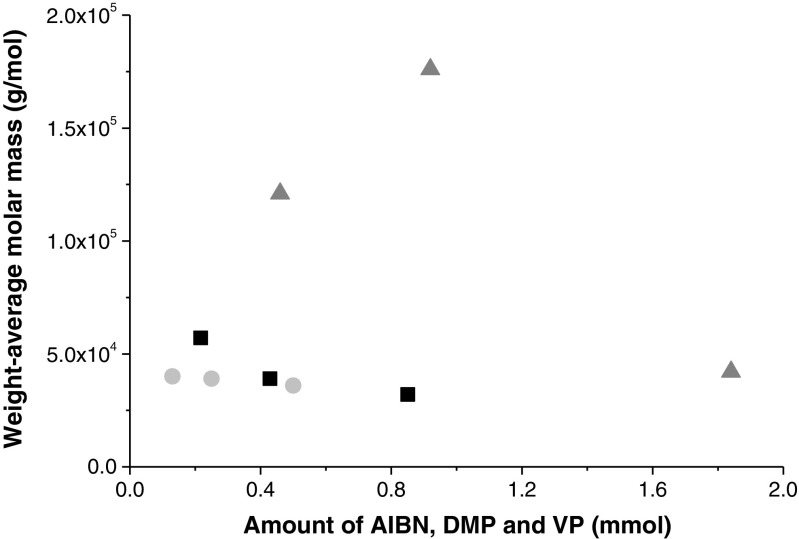
Changes in the M_w_ of PVP-CTA during the first radical reaction. Weight-average molar mass (g/mol) versus the amounts (mmol) of AIBN (■), DMP (), and VP ()

Figure 5 shows the change in M_w_ when three different amounts of AIBN, DMP, and VP were used in the first radical reaction (MPC-4 to MPC-23), which yields PVP-CTA. It is possible to observe that M_w_ decreases with an increasing amount of AIBN (■) and when DMP () is varied, there was no remarkable variation in M_w_. Thus, DMP had no effect on M_w_ in the investigated range. There appears to be no clear relationship between M_w_ and the amount of VP () although rather large differences in M_w_ were observed. Moreover, M_w_ was almost the same when 75.6 mmol or 1.84 mmol of VP was used (see Tables 1 and 3; MPC-4 and MPC-20, respectively), which could mean that 1.84 mmol is the minimal amount of VP needed to enable the synthesis of the polymer.

**Fig. 6:**
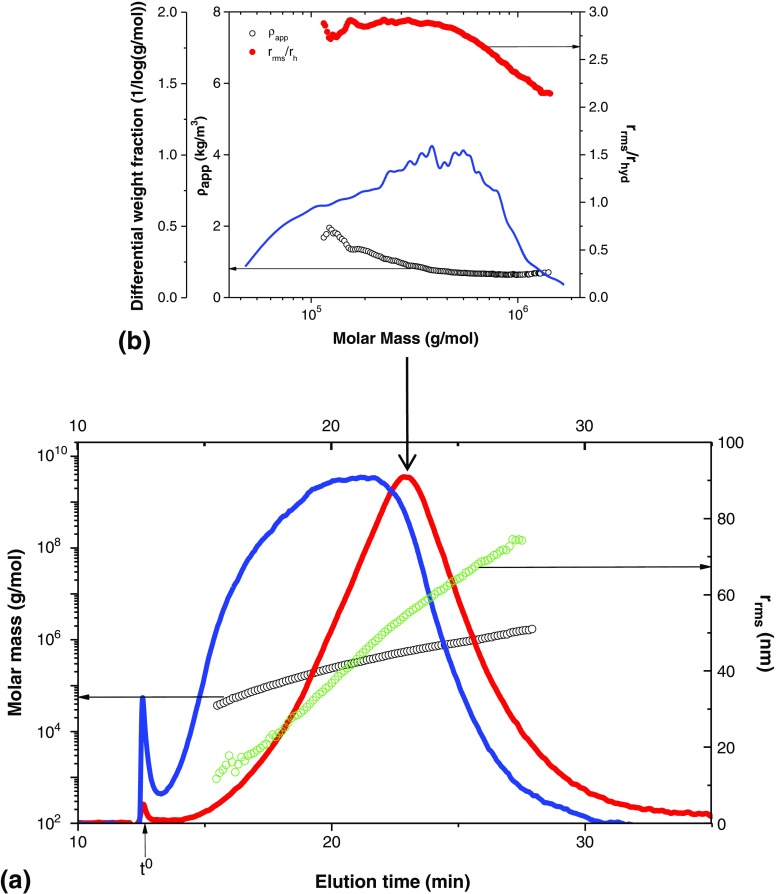
AF4 fractogram for sample MPC-24. (**a**) Molar mass distribution M (○), root- mean-square radius distribution r_rms_ () Rayleigh ratio from MALS at 90^0^ () and differential refractive index dRI () versus the elution time (min). (**b**) Differential weight fraction 1/log(g/mol) (), apparent density ρ_app_ (○) and the ratio between the root- mean-square radius r_rms_ and hydrodynamic radius r_hyd_ as a conformational parameter versus molar mass (g/mol)

A representative fractogram of sample MPC- 24 is shown in Fig. 6. For all samples from the second radical reaction (MPC-24 to MPC-32), which yields PVP-b-PAMPS, only one population was observed. This population (elution times of approximately 19–28 min, Fig. 6a) displays a rod-like conformation (r_rms_/r_hyd_ = 2.1–2.8, see Fig. 6b) [20].

**Fig. 7:**
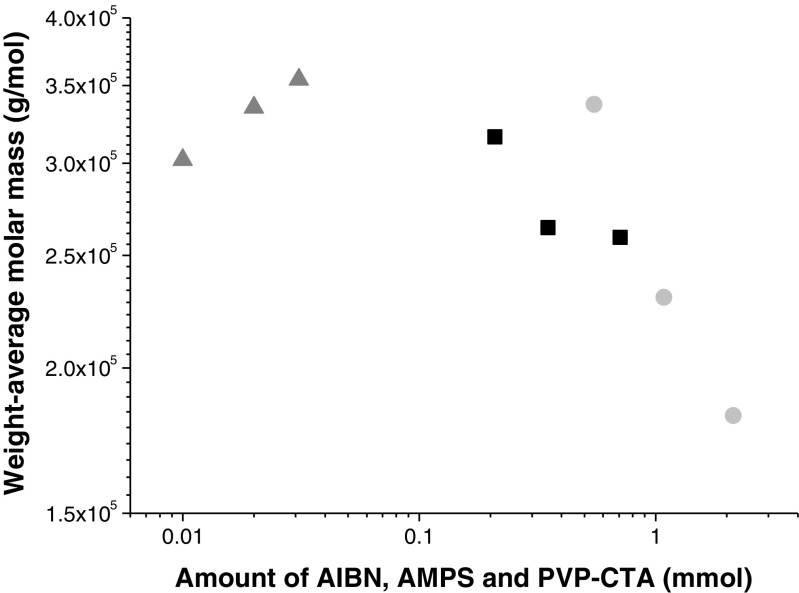
Changes in the M_w_ of PVP-b-PAMPS during the second radical reaction. Weight-average molar mass (g/mol) versus the amounts of AIBN (■), AMPS (), and PVP-CTA ()

Figure 7 shows the M_w_ that resulted from of the second radical reaction when the amounts of AIBN, AMPS, and PVP-CTA were varied. A lower amount of AIBN (■) resulted in a slightly higher M_w_. Increases in the amount of AMPS () caused a decrease in M_w_, whereas increasing the amount of PVP-CTA () caused an increase in M_w_ which means that the M_w_ is regulated by the addition of both reactants (AIBN and PVP-CTA). It can be noticed that PVP-CTA seems to be the limiting reagent of the M_w_. A considerable increase in AMPS (0.55 mmol to 21.86 mmol, see Tables 1 and 3; MPC- 24 and MPC-28, respectively) did not lead to a decrease in M_w_, as expected according to the results shown in Fig. 7. The fact that M_w_ remained similar may have been due to the increase in PVP-CTA (0.016 mmol to 0.02 mmol; see Table 1; MPC-24 and MPC-28, respectively).

## Conclusion

In this study, the change in M_w_ when the amounts of the reactants were varied during each radical reaction, which yielded PVP-CTA and PVP-b-PAMPS, was determined using AF4-MALS-dRI. For the first radical reaction, PVP-CTA was separated in two populations by AF4. The first population displayed an elongated, rod-like or random coil conformation, and the second population had a very different conformation, which scaled constantly in relation to M, potentially suggesting the presence of supramolecular aggregates. The presence of supramolecular aggregates has, to the best of our knowledge, not been reported previously in other studies on the same polymer. Additionally, it was found that different M_w_ values of PVP-CTA could be obtained by manipulating the amount of AIBN, whereas DMP did not have a large effect on M_w_. In addition, the variation in the amount of VP showed no clear relationship with M_w_.

For the second radical reaction, only one population with a rod-like conformation was found. Moreover, higher amounts of AIBN and AMPS could produce a decrease in M_w_, whereas varying the amount of PVP-CTA caused an increase in M_w_. It seems that PVP-CTA could be the limiting reagent for the synthesis of PVP-b-PAMPS. Finally, it was shown that, using AF4, it was possible to obtain detailed information regarding PVP-CTA and PVP-b-PAMPS.

## References

[1] Luo Y-L, Yuan J-F, Liu X-J, Xie H, Gao Q-Y (2010). Self-assembled polyion complex micelles based on PVP-b-PAMPS and PVP-b-PDMAEMA for drug delivery. J Bioactive Compatible Polym.

[2] Barros JAG, Fechine GJM, Alcantara MR, Catalani LH (2006). Poly(*N*-vinyl-2-pyrrolidone) hydrogels produced by Fenton reaction. Polymer.

[3] Xu J, Pelton R (2004). A new route to poly(*N*-isopropylacrylamide) microgels supporting a polyvinylamine corona. J Colloid Interface Sci.

[4] Luo Y-L, Yuan J-F, Shi J-H, Gao Q-Y (2010). Synthesis and characterization of polyion complex micelles and their controlled release of folic acid. J Colloid Interface Sci.

[5] Auriemma F, De Rosa C, Di Girolamo R, Malafronte A, Scoti M, Mitchell GR, Esposito S, Mitchell GR, Tojeira A (2016). Relationship between molecular configuration and stress-induced phase transitions. Controlling the morphology of polymers: multiple scales of structure and processing.

[6] Žigon M, The NK, Cheng S, Grubišić-Gallot Z (1997). Degradation of high molecular weight polystyrenes during the SEC separation process, as demonstrated by SEC coupled with lalls and by static light scattering. J Liquid Chromatogr Relat Technol.

[7] Barth HG (1982). High-performance size-exclusion chromatography of hydrolyzed plant proteins. Anal Biochem.

[8] Otte T, Pasch H, Macko T, Brüll R, Stadler FJ, Kaschta J, Becker F, Buback M (2011). Characterization of branched ultrahigh molar mass polymers by asymmetrical flow field-flow fractionation and size exclusion chromatography. J Chromatogr A.

[9] Cave RA, Seabrook SA, Gidley MJ, Gilbert RG (2009). Characterization of starch by size-exclusion chromatography: the limitations imposed by shear scission. Biomacromolecules.

[10] Wahlund KG, Giddings JC (1987). Properties of an asymmetrical flow field-flow fractionation channel having one permeable wall. Anal Chem.

[11] Litzén A, Wahlund KG (1991). Zone broadening and dilution in rectangular and trapezoidal asymmetrical flow field-flow fractionation channels. Anal Chem.

[12] Wahlund K-G, Nilsson L, SKR W, Caldwell KD (2012). Flow FFF – basics and key applications. Field-flow fractionation in biopolymer analysis.

[13] Nilsson L (2013). Separation and characterization of food macromolecules using field-flow fractionation: a review. Food Hydrocolloids.

[14] Malik MI, Pasch H (2016). Field-flow fractionation: new and exciting perspectives in polymer analysis. Prog Polym Sci.

[15] Håkansson A, Magnusson E, Bergenståhl B, Nilsson L (2012). Hydrodynamic radius determination with asymmetrical flow field-flow fractionation using decaying cross-flows. Part I. A theoretical approach. J Chromatogr A.

[16] Magnusson E, Håkansson A, Janiak J, Bergenståhl B, Nilsson L (2012). Hydrodynamic radius determination with asymmetrical flow field-flow fractionation using decaying cross-flows. Part II. Experimental evaluation. J Chromatogr A.

[17] Berry GC (1966). Thermodynamic and conformational properties of polystyrene. I. Light-scattering studies on dilute solutions of linear polystyrenes. J Chem Phys.

[18] Andersson M, Wittgren B, Wahlund K-G (2003). Accuracy in multiangle light scattering measurements for molar mass and radius estimations. Model calculations and experiments. Anal Chem.

[19] Nilsson L, Leeman M, Wahlund K-G, Bergenståhl B (2006). Mechanical degradation and changes in conformation of hydrophobically modified starch. Biomacromolecules.

[20] Burchard W, Roovers J (1999). Solution properties of branched macromolecules. Branched polymers, II.

[21] Schmidt M, Nerger D, Burchard W (1979). Quasi-elastic light scattering from branched polymers: 1. Polyvinylacetate and polyvinylacetate—microgels prepared by emulsion polymerization. Polymer.

